# Changing outcomes in patients with chronic liver disease in intensive care: a decade of experience

**DOI:** 10.1186/cc11000

**Published:** 2012-03-20

**Authors:** MJ McPhail, DL Shawcross, RD Abeles, G Huei-Lee, M Abdulla, T Chang, C Willars, E Sizer, G Auzinger, W Bernal, J Wendon

**Affiliations:** 1 King's College Hospital, London, UK

## Introduction

Patients with chronic liver disease requiring intensive care are thought to carry a poor prognosis in comparison with noncirrhotic patients with similar severity of illness. During the last decade improvements in multiple areas of management in patients in the ICU have occurred but improvement in outcomes in patients with cirrhosis has not been shown.

## Methods

Between 1 January 2000 and 31 December 2010 all patients admitted to the Liver Intensive Therapy Unit (LITU) at King's College Hospital had daily prospective collection of demographic, biochemistry and bedside physiology. These data were used to quantify the severity of illness (APACHE II and Model for End-stage Liver Disease (MELD)) and outcomes in these patients.

## Results

A total of 958 patients (median age 52 (range 16 to 90) years; 603 (62%) male) with cirrhosis and emergency LITU admission were identified. Aetiology of cirrhosis was alcohol in 43%, viral in 10%, autoimmune disease in 10% and nonalcoholic fatty liver disease/metabolic in the remainder. The pattern of aetiology of cirrhosis did not change over time and a viral aetiology was associated with improved outcome (OR 0.53, 95% CI 0.34 to 0.81, *P *= 0.003); alcohol was not associated with poorer outcome (*P *= 0.09). The primary reasons for admission were bleeding (33%), sepsis (27%), hepatic encephalopathy (17%), metabolic (7%) and other (16%). The median APACHE II score was 21 (5 to 50) and the median MELD score 23 (3 to 50). Overall LITU survival was 63% and survival to hospital discharge 51%. LITU survival increased from 47% to 73% over the study period (2000 to 2010) with hospital outcome improving from 40% to 63%. The median admission APACHE II score fell from 23.4 to 21.9 over the study period (*P *< 0.001) with the MELD score falling from 23.4 to 18.3 (*P *< 0.001). Length of LITU stay did not change significantly over the study period (*P *= 0.092). The reduction in illness severity was predominantly due to a smaller percentage of patients with renal failure and those with three or more organs in failure (32% up to 2005 and 24% post 2005, *P *= 0.004). The reduction in MELD score related to decreased renal dysfunction; creatinine over the study period (1.9 mg/dl to 1.6 mg/dl, *P *< 0.001) with no change in bilirubin, and by contrast a small rise in international normalised ratio (INR 1.8 to 2.2, *P *= 0.07).

## Conclusion

Survival of patients with cirrhosis admitted to the specialist LITU is improving over time. The factors relating to this may be resultant upon earlier admission to critical care and a lower incidence of renal dysfunction. Alcohol aetiology is not relevant to outcome.

